# Congenic Mice Confirm That Collagen X Is Required for Proper Hematopoietic Development

**DOI:** 10.1371/journal.pone.0009518

**Published:** 2010-03-03

**Authors:** Elizabeth Sweeney, Douglas Roberts, Tina Corbo, Olena Jacenko

**Affiliations:** Department of Animal Biology, School of Veterinary Medicine, University of Pennsylvania, Philadelphia, Pennsylvania, United States of America; Katholieke Universiteit Leuven, Belgium

## Abstract

The link between endochondral skeletal development and hematopoiesis in the marrow was established in the collagen X transgenic (Tg) and null (KO) mice. Disrupted function of collagen X, a major hypertrophic cartilage matrix protein, resulted in skeletal and hematopoietic defects in endochondrally derived tissues. Manifestation of the disease phenotype was variable, ranging from perinatal lethality in a subset of mice, to altered lymphopoiesis and impaired immunity in the surviving mice. To exclude contribution of strain specific modifiers to this variable manifestation of the skeleto-hematopoietic phenotype, C57Bl/6 and DBA/2J collagen X congenic lines were established. Comparable disease manifestations confirmed that the skeleto-hematopoietic alterations are an inherent outcome of disrupted collagen X function. Further, colony forming cell assays, complete blood count analysis, serum antibody ELISA, and organ outgrowth studies established altered lymphopoiesis in all collagen X Tg and KO mice and implicated opportunistic infection as a contributor to the severe disease phenotype. These data support a model where endochondral ossification-specific collagen X contributes to the establishment of a hematopoietic niche at the chondro-osseous junction.

## Introduction

In vertebrates, the formation of a hematopoietic marrow within bone is intimately coordinated with the endochondral mechanism of skeletal development [1], [2]. During embryogenesis, hematopoiesis is sequentially re-established first in the yolk sac, then liver, spleen, and finally marrow, which remains the predominant site of blood cell production after birth [3]. Through use of mouse models that express an altered endochondral ossification (EO)-specific extracellular matrix (ECM) protein, collagen X, hematopoiesis and immune function have been linked to endochondral skeletogenesis [4], [5], [6], [7], [8].

As EO initiates during embryogenesis, the future axial and appendicular skeleton, as well as certain cranial bones are first represented as a cartilaginous blueprint [1], [9]. These cartilage primordia allow for rapid tissue growth, and identify future skeletal regions where a marrow could form. The eventual replacement of the cartilaginous anlagen by bone and marrow relies on the sequential maturation of chondrocytes to hypertrophy. Chondrocyte hypertrophy results in an increase in cell size and synthesis of a unique ECM consisting predominantly of collagen X. Through the combined effects of the hypertrophic cartilage matrix components and a repertoire of growth and signaling factors, there is vascular invasion and influx of mesenchymal cells, hematopoietic precursors, and osteo/chondroclasts into this primary ossification center. As the hypertrophic cartilage begins to be degraded, matrix remnants serve as scaffolds upon which osteoblasts deposit osteoid, thus forming trabecular bony spicules that protrude into the newly forming marrow. Continual replacement of hypertrophic cartilage, together with establishment of secondary ossification centers at outer (epiphyseal) tissue ends, defines the cartilaginous growth plates that provide bones with longitudinal growth potential until maturity. This chondro-osseous junction, consisting of the hypertrophic cartilage layer of the growth plate and trabecular bone, undergoes constant remodeling during growth and is a site where blood cells can colonize spaces carved out from the embryonic cartilage.

The link between EO and hematopoiesis was first suggested by the disease phenotype of the collagen X mouse models, where collagen X function in the growth plate was disrupted either by transgenesis (Tg mice; [5], [6], [10], [11]), or through gene inactivation (KO mice; [7], [12]). The Tg mice were generated using different lengths (4.7 or 1.6 kb) of the chicken collagen X promoter to express in hypertrophic cartilage [6] collagen X with truncations within the central triple-helical domain (e.g. lines: 1.6–293Δ and 4.7–21Δ used in this study). Similar skeletal and hematopoietic disease phenotypes were observed in the multiple resultant Tg lines, each with an independent transgene insertion site(s), thus eliminating the effect of transgene insertional mutagenesis towards the disease phenotype [6], [10]. Additionally, extra-skeletal presence of either the transgene or endogenous collagen X was excluded by RT-PCR with species-specific primers, confirming that collagen X is not expressed in brain, eye, heart, kidney, liver, lung, muscle, skin, spleen, thymus, and marrow [13]. These observations were further confirmed by northern blot analysis, *in situ* hybridization, and immunohistochemistry [13]. Together, these approaches implied that the skeletal and hematopoietic changes in the collagen X Tg and KO mice might directly ensue from disruption of collagen X function in growth plates [1], [5], [6], [7], [8], [11], [14].

The goals of this study were to address the cause of the variable disease phenotype within Tg and KO mouse lines, first by excluding the potential contribution of strain specific loci modifications, which in the presence of disrupted collagen X might contribute to phenotypic variability [15]. Having already ruled out other potential causes [13], an exclusion of genetic background influences would directly implicate collagen X disruption within the chondro-osseous junction as the underlying factor of all hematopoietic and immune response changes in the mice. For this purpose, congenic collagen X Tg and KO lines were established by inbreeding mice to the C57BL/6 and DBA/2J strains to yield 99.98% homogeneity to one strain [16]. Comparison of the murine disease phenotype in the congenic mice to the outbred collagen X Tg and KO mice via gross outward changes, body and organ measurements, histology, and flow cytometry led to the exclusion of strain specific modifiers as contributors to phenotypic variability. Further analysis of these mouse strains via organ culture assays, complete blood cell analysis, flow cytometry and colony forming cell assays implied that perinatal lethality in the collagen X Tg and KO mice results from lymphopenia coupled to opportunistic infections. Taken together, these data implicate the highly orchestrated events of EO, involving the collagen X matrix within the chondro-osseous junction, in contributing towards the establishment of a hematopoietic niche that is prerequisite for proper lymphopoiesis.

## Results

### Phenotypic Variability Persists in Congenic Strains of Collagen X Tg and KO Mice

Strain specific modifiers have contributed to the disease phenotype of several transgenic mouse models [15], which was refined following inbreeding onto a pure background strain. To ensure that the disruption of collagen X function rather than presence of strain specific modifiers caused the variable skeleto-hematopoietic phenotype in the collagen X Tg and KO mice, we generated congenic Tg and KO lines by backcrossing greater then twelve generations into C57BL/6 and DBA/2J strains. Such inbreeding ensured an essentially isogenic background (99.98% identical) with that strain, except for the chromosomal segment carrying the transgene/knocked-out gene [16]. The effect of inbreeding on disease phenotype variability was first assessed by determining the percentage of perinatal lethality around week-3 in four congenic collagen X Tg lines (1.6–293Δ B6, 1.6–293Δ DBA, 4.7–21Δ B6 and 4.7–21Δ DBA) and two KO lines (KO B6 and KO DBA) as compared to outbred Tg and KO lines (a C57BL/6, SJL, 129Sv and DBA/2J mix). Comparable perinatal lethality ratios were observed across all congenic and outbred strains (e.g. a range of 15.7–18.7% for 1.6–293Δ Tg line, 18.8–25.6% for 4.7–21Δ Tg line, and 7.4–10.6% for KO) (**Table 1**), suggesting that variability in disease severity was an inherent outcome of collagen X disruption. Additionally, the surviving congenic collagen X Tg mice continued to exhibited transient dwarfism, as was originally described in outbred Tg mice [5], [7], which manifests as a ∼21% reduction in body weight of outbred and congenic Tg mice at week-3, as compared to controls (**Table 2**). Body weight of surviving KO mice, however, did not deviate substantially from that of controls in any strain. In contrast, ∼50% reduction in overall body size and weight was observed in the perinatal lethal subset of both the outbred and congenic collagen X Tg and KO mice, when compared to controls (**Table 2**).

**Table 1:** Percent perinatal lethality in collagen X outbred and congenic mice.

	OUTBRED	C57BL/6	DBA/2J
**1.6**–**293Δ**	18.7% (n = 7641)	15.7% (n = 1045)	17.1% (n = 1264)
**4.7**–**21Δ**	25.55% (n = 8204)	18.8% (n = 2620)	24.2% (n = 1641)
**KO**	10.6% (n = 1595)	7.4% (n = 448)	7.5% (n = 252)

Conservation of perinatal lethality for the outbred and congenic (C57Bl/6 and DBA/2J) collagen X transgenic mice (1.6–293Δ and 4.7–21Δ) and collagen X null (KO) mice excluded contribution of a strain-specific modifier to the variable disease phenotype. Percent perinatal lethality was calculated as the number of wasting mice around week-3 (n) divided by the total number of weaned offspring given by mutant producing parents.

**Table 2:** Comparison of organ weights from outbred and congenic collagen X mice.

Organ Weights						
	Outbred WT	C57BL/6	DBA/2J			
Body	9.24±0.22 (15)	8.81±0.21 (43)	8.15±0.25 (48)			
Kidney	72.10±1.76 (16)	61.42±1.28 (43)	64.60±2.06 (50)			
Kidney:Body	128∶1 (15)	143∶1±2 (43)	128∶1±1 (48)			
Spleen	79.33±7.44 (10)	61.76±2.78 (43)	75.68±3.64 (50)			
Thymus	37.31±1.46 (15)	55.72±1.42 (43)	38.97±1.37 (50)			
Kidney:Spleen	0.90∶1 (10)	0.99∶1±0.04 (43)	1.14∶1±0.04 (50)			
Kidney:Thymus	0.52∶1 (15)	0.91∶1±0.02 (43)	0.60∶1±0.01 (50)			
	**Outbred Tg**	**C57BL/6 Tg**	**DBA/2J Tg**	**Outbred Tg severe**	**C57/Bl6 Tg severe**	**DBA/2J Tg severe**
Body	7.34±0.31 (7)	6.64±0.22 (21)	6.85±0.22 (19)	4.74±0.14 (45)	3.47±0.36 (5)	3.81±0.16 (8)
Kidney	56.09±2.32 (7)	48.3±1.6 (21)	52.64±1.42 (19)	38.49±1.05 (46)	25.74±4.48 (5)	33.56±2.82 (8)
Kidney:Body	131∶1 (7)	138∶1±2 (21)	130∶1±2 (19)	123∶1 (45)	140∶1±11 (5)	117∶1±6 (8)
Spleen	31.38±2.63 (7)	30.8±2.4 (21)	50.35±3.24 (19)	9.06±0.50 (47)	6.74±0.75 (5)	9.33±1.51 (8)
Thymus	37.06±1.89 (7)	34.7±1 (21)	32.74±1.43 (19)	8.50±0.68 (47)	3.42±0.58 (5)	7.98±1.60 (8)
Kidney:Spleen	0.57∶1 (7)	0.62∶1±0.03 (21)	1.14∶1±0.04 (19)	0.23∶1 (46)	0.29∶1±0.06 (5)	0.28∶1±0.04 (8)
Kidney:Thymus	0.66∶1 (7)	0.73∶1±0.02 (21)	1.24∶1±0.13 (19)	0.22∶1 (46)	0.14∶1±0.02 (5)	0.24∶1±0.04 (8)
	**Outbred KO**	**C57BL/6 KO**	**DBA/2J KO**	**Outbred KO severe**	**C57/Bl6 KO severe**	**DBA/2J Tg severe**
Body	8.41±0.17 (33)	8.68±0.20 (50)	8.68±0.17 (50)	5.40±0.27 (10)	3.96±0.30 (4)	3.58 (1)
Kidney	61.66±1.60 (41)	58.74±1.17 (50)	63.16±1.35 (50)	45.69±2.35 (13)	31.98±1.97 (4)	40.70±14.50 (2)
Kidney:Body	141∶1 (33)	148∶1±2 (50)	138∶1±1 (50)	118∶1 (10)	123∶1±4 (4)	137∶1 (1)
Spleen	58.73±3.71 (44)	64.38±2.62 (50)	77.41±2.91 (50)	10.12±1.12 (10)	9.43±1.41 (4)	4.3 (1)
Thymus	48.58±2.23 (40)	59.41±1.23 (50)	45.37±1.15 (50)	8.52±1.35 (12)	6.88±1.14 (4)	5.55±0.45 (2)
Kidney:Spleen	0.95∶1 (41)	1.09∶1±0.04 (50)	1.21∶1±0.05 (50)	0.22∶1 (10)	0.29∶1±0.03 (4)	0.164∶1 (1)
Kidney:Thymus	0.79∶1 (40)	1.01∶1±0.01 (50)	0.72∶1±0.01 (50)	0.19∶1 (12)	0.21∶1±0.03 (4)	0.152∶1 (2)

Body and organ weights of spleen, both thymic lobes, and the left kidney from outbred and congenic (C57Bl/6 and DBA/2J) control (WT), collagen X transgenic (Tg), null (KO), and perinatal lethal (Tg severe or KO severe) mice show conservation of weight trends across all strains. Numbers in parenthesis indicate the number of animals analyzed.

Comparison of organ weights revealed a similar trend in outbred and congenic lines. For these analyses, kidney weights were used to normalize for body size when comparing lymphatic organs since the kidney to body ratios were similar among all mouse lines. Normalization revealed a reduction of spleen size in all Tg lines when compared to controls (**Table 2**: kidney:spleen). Moreover, both outbred and congenic perinatal lethal Tg and KO mice had severely diminished spleens and thymuses, when compared to controls (**Table 2**: kidney:spleen; kidney:thymus; individual organ weights).

### Altered Chondro-Osseous Junction, Marrow Cellularity and Decreased B Lymphopoiesis in Collagen X Tg and KO Mice

**Figure 1:**
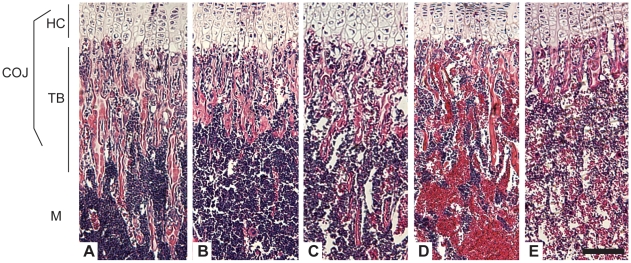
Collagen X Tg and KO mice have an altered chondro-osseous junction. Hematoxylin and eosin staining of longitudinal sections of tibia from (A) week-3 C57Bl/6 wild type (WT), (B) C57Bl/6 congenic collagen X transgenic (Tg), (C) null (KO), and perinatal lethal (D) Tg, and (E) KO. The chondro-osseous junction (COJ) is shown, including hypertrophic cartilage (HC), trabecular bone (TB), and marrow (M). Note diminished trabecular bony spicules in all collagen X Tg and KO mice, with the greatest reductions in (D) and (E), with a concomitant increase in erythrocytes in the marrow. Bar = 150 µm.

Hematoxylin and eosin staining of the chondro-osseous junction, the intersection of growth plate hypertrophic chondrocytes and the newly forming trabecular bone and marrow, of the congenic collagen X Tg and KO mice were comparable to the changes observed in the outbred collagen X Tg and KO mice (**Fig. 1**) [5]. Specifically, in both Tg and KO mice, alterations within the cartilage growth plate zones persisted [5], [7] (data not shown). Moreover, in both Tg and KO mice (**Fig. 1B, C**), trabecular bone spicules, comprised of hypertrophic cartilage cores and newly deposited bone, were reduced, especially in perinatal lethal subset (**Fig. 1D, E**). While these changes were most prominent in rapidly-growing week-3 mice, diminished trabecular bone and generalized osteopenia persisted throughout life [**5**]. These defects were seen in all EO-derived axial, appendicular, and cranial skeletal elements [6], [17].

**Figure 2:**
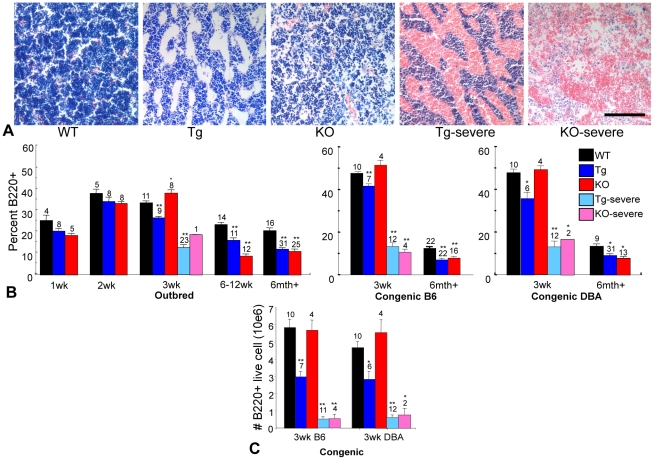
Collagen X Tg and KO mice have altered bone marrow cellularity and lymphocyte profiles. (A) Geimsa staining of longitudinal sections of tibia from week-3 C57Bl/6 wild type (WT) and C57Bl/6 congenic collagen X transgenic (Tg), null (KO), and perinatal lethal (Tg severe, KO severe) mice showed mild hyperplasia in Tg and KO and aplasia with an influx of red blood cells in Tg severe and KO severe. (40X) Bar = 125 µm. (B) Temporal analysis by flow cytometry for B220^+^ B lymphocytes in bone marrow aspirates of outbred and congenic (C57Bl/6, B6 and DBA/2J, DBA) mice revealed reduced B220^+^ lymphocytes throughout life in Tg and KO, with an exception at week-3 in KO, in both the outbred and congenic strains. Note also acute B cell reduction in Tg severe and KO severe. (C) The total number of B220^+^ lymphocytes was calculated via flow cytometry and the trend of B lymphocyte reduction in the collagen X Tg and KO mice is maintained. Numbers above the standard error of the mean are the number of mice per group. (*p<0.05, **p<0.01)

Changes in marrow cellularity of collagen X Tg and KO mice were detectable both histologically and by flow cytometry. Giemsa staining of mouse marrows revealed a subtle decrease in cell density in Tg and KO congenic collagen X samples, apparent as presence of more open spaces (**Fig. 2A**). Leukocyte reduction, characteristic of marrow hypoplasia, was accompanied by dramatic erythrocyte predominance in the Tg and KO perinatal lethal mouse marrows, as was also observed in the outbred perinatal lethal mice (**Fig. 2A**) [5], [7]. These changes were readily apparent upon skeletal dissection due to the dark red marrow in all EO-derived bones [7]. Further, flow cytometry confirmed altered B lymphopoiesis, which revealed diminished B220^+^, CD138^+^, IgM^+^, and IgD^+^ lymphocytes from marrows of all outbred and congenic collagen X Tg and KO mice throughout life, with most dramatic reductions in the perinatal lethal mice (**Fig. 2B** and [5]). The one exception was a transient increase of B220^+^ B lymphocytes at week-3 in the outbred and congenic collagen X KO mice (**Fig. 2B**). The overall reduction of B lymphocytes in the collagen X Tg and KO mice was maintained when the number of live B220^+^ cells were analyzed, confirming that the alteration in B lymphocyte percentage was not an artifact of the total number of nucleated cells analyzed, for example the perinatal lethal subset which has a dramatic reduction in total leukocytes (**Fig. 1A**, **2A, C**).

### Altered Lymphatic Organ Architecture and Lymphocyte Profiles in Collagen X Tg and KO Mice

**Figure 3:**
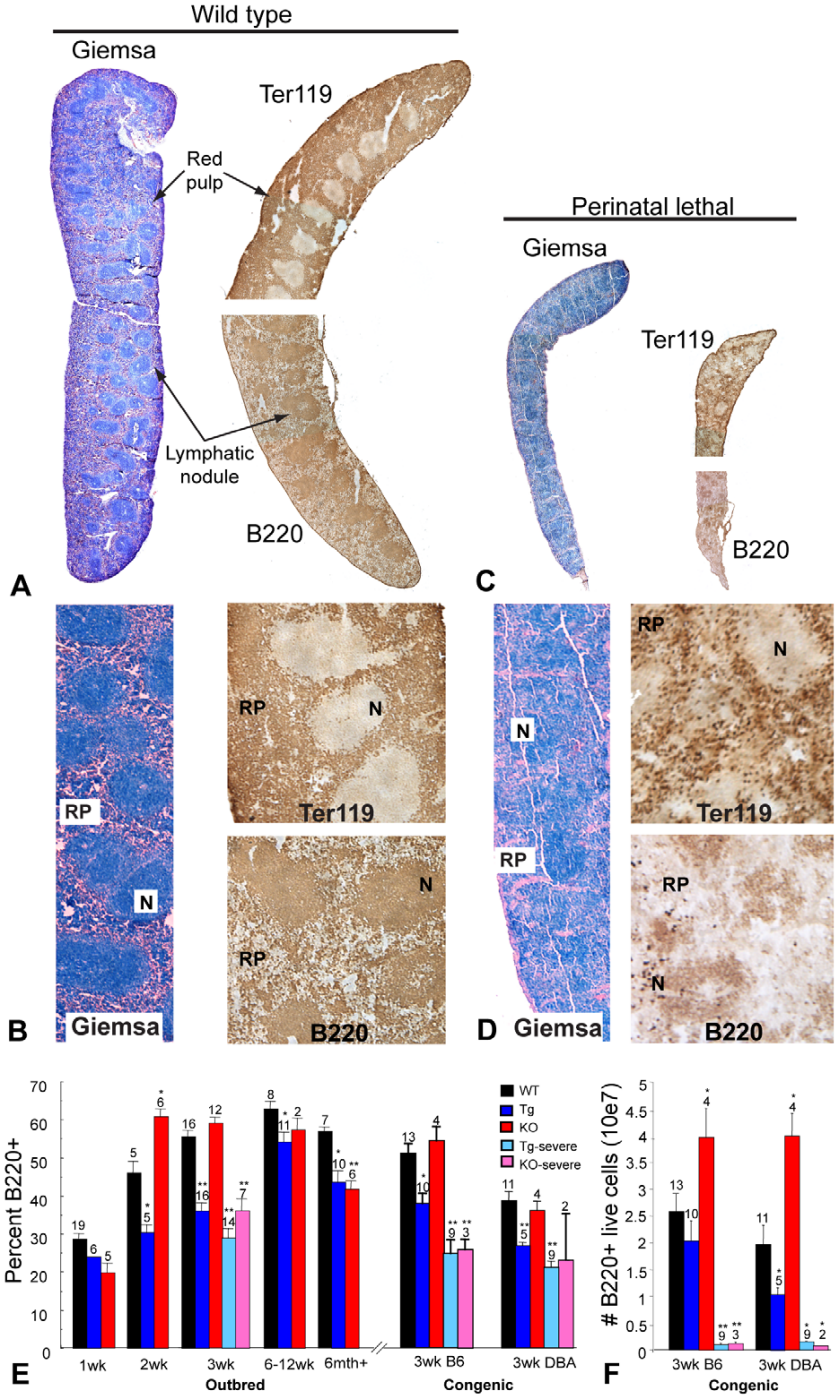
Altered spleen architecture and B lymphocyte profile in the collagen X Tg and KO mice. Giemsa and immunohistochemical staining of spleens from week-3 C57Bl/6 wild type (A, 2X; B, Hi mag) and congenic collagen X perinatal lethal mice (C, 2X; D, Hi mag) revealed diminished organs with altered tissue architecture in the perinatal lethal mice. Staining for red blood cells with TER119 antibody (upper halves of spleens on right of Giemsa stained sections) showed decreased zones of red pulp (RP) in perinatal lethal mouse spleen. Staining for B lymphocytes with B220 antibody (lower halves of spleens on right of Giemsa stained sections) revealed diffuse B220^+^ staining and a reduction of lymphatic node (N) size in perinatal lethal mouse spleen. Bar = 25 mm. (E) Temporal analysis by flow cytometry for B220^+^ B lymphocytes from of outbred and congenic (C57Bl/6, B6 and DBA/2J, DBA) wild type (WT) and collagen X transgenic (Tg), null (KO), and perinatal lethal (Tg severe, KO severe) mouse spleens revealed decreased B lymphocytes throughout life, with the exception of KO at weeks two and three, in both the outbred and congenic strains. (F) Number of B220^+^ B lymphocytes in congenic WT, collagen X Tg and KO mice. Note depletion of B lymphocytes in Tg severe and KO severe. Numbers above the standard error of the mean are the number of mice per group. (*p<0.05, **p<0.01)

Lymphatic organs of perinatal lethal congenic mice depicted gross, histological, as well as hematopoietic changes. Spleens and thymuses were diminished in size compared to those of wild type mice (**Table 2**) and lymph nodes were undetectable. Spleens were also discolored upon visual inspection and exhibited altered architecture upon histological analyses consisting of a markedly diminished red pulp and poorly defined lymphatic nodules (**Fig. 3A-D**). Immunostaining for red blood cells (Ter119) and B lymphocytes (B220) confirmed decreased cellularity in the red pulp and lymphatic nodules (**Fig. 3A-D**). Additionally, flow cytometry of splenocytes from outbred and congenic collagen X Tg and KO mice confirmed depletion of B220^+^ lymphocyte throughout life, with most dramatic reductions measured in the moribund perinatal lethal mice at week-3 (**Fig. 3E, F**). Interestingly, when compared to wild type at weeks-2 and -3, the collagen X KO mice have increased levels of B220^+^ splenocytes, mirroring the profile reported for marrow in KO mice (**Figs. 2**, **3E, F**). Again, the overall trend of diminished live B220^+^ cell numbers was maintained when total number of positive cells was calculated (**Fig. 3F**).

**Figure 4:**
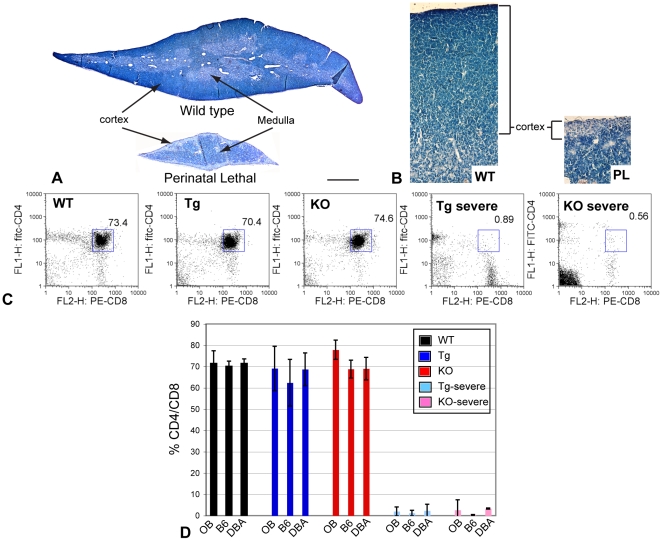
Diminished number of immature thymocytes and altered thymic architecture in the collagen X perinatal lethal mice. (A, B) Giemsa staining of thymuses from week-3 wild type (WT) and collagen X transgenic perinatal lethal (PL) mice reveals a reduction in size and depletion of the cortical thymocytes and region in the perinatal lethal mouse thymus, (2X) Bar = 775 µm and 20X respectively. (C) Flow cytometry analysis of week-3 C57Bl/6 wild type, congenic collagen X transgenic (Tg), null (KO) and perinatal lethal (Tg severe and KO severe) mouse thymocytes quantifies the percent double-positive, CD4^+^/CD8^+^, immature T lymphocytes (boxed). (D) Graphic representation of compiled flow cytometry data reveals decreased levels of CD4^+^/CD8^+^ immature T lymphocytes in perinatal lethal collagen X Tg and KO mice across all strains (outbred; OB, C57Bl/6; B6, and DBA/2J; DBA). n≥6 mice per group.

Pronounced architecture and cell content changes were also evident in thymuses from congenic Tg and KO perinatal lethal mice. Histology not only emphasized the reduction in thymic size, but revealed comparable changes in structure and cellularity seen in the outbred perinatal lethal mice (**Fig. 4A, B**) [5], [7]. Specifically, lack of what should have been an extensive and densely populated cortex in the collagen X perinatal lethal mouse thymus suggested a depletion of marrow-derived, immature T lymphocytes; the medulla, however, still maintained a mature T lymphocyte population, possibly originating from fetal liver hematopoiesis [18] (**Fig. 4B**). Depletion of the immature T lymphocyte population was confirmed by flow cytometry analysis of wild type, outbred collagen X and congenic collagen X mouse thymocytes, revealing a dramatic decrease in CD4^+^/CD8^+^ immature T cells in the perinatal lethal mice (**Fig. 4C, D**). Taken together, these data are consistent with the disruption of collagen X function in hypertrophic cartilage, and not mouse strain specific modifiers, as being responsible for an altered marrow environment and the peripheral lymphatic alterations changes observed in the collagen X Tg and KO mice.

### Colony Forming Cell Assays Confirm Altered Hematopoiesis in the Collagen X Tg and KO Mice

Colony forming cell (CFC) assays further revealed the changes in hematopoietic cell differentiation in all collagen X Tg and KO mice. These assays were performed separately for each outbred and congenic line, and the results continued to be interchangeable; thus, C57Bl/6 strains or pooled data from all strains will be presented for the remainder of this study.

**Figure 5:**
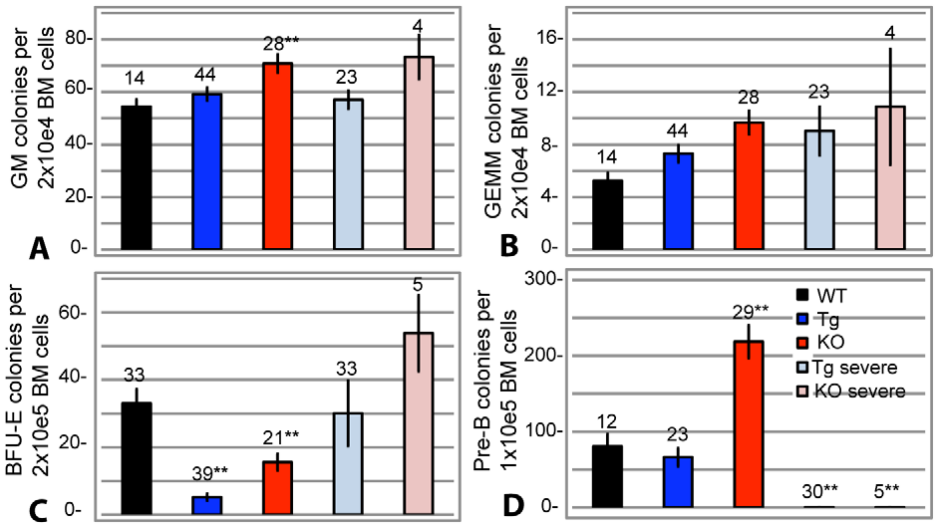
Colony forming cell assays confirm altered hematopoietic lineage commitment in the collagen X Tg and KO mice. Colony forming assays using marrow aspirates from week-3 wild type (WT), collagen X transgenic (Tg), null (KO) and perinatal lethal (Tg severe and KO severe) mice. Cells cultured in: (A-B) complete Methocult media to enumerate granulocyte-macrophage (GM) and granulocyte-erythrocyte-monocyte-macrophage (GEMM) colonies; (C) erythropoietin supplemented Methocult media to enumerate pre-erythrocyte blast colonies (BFU-E); (D) interluekin-7 supplemented Methocult media to enumerate pre-B lymphocyte colonies (pre-B). Numbers above the standard error of the mean are number of samples. (*p<0.05, **p<0.001).

The CFC assays quantify multi-potential and committed blood cells in the marrow by measuring the formation of cell specific colonies, e.g. granulocyte-macrophage (GM), granulocyte-erythrocyte-monocyte-macrophage (GEMM), erythrocyte blast forming unit (BFU-E), and pre-B lymphocytes (pre-B). There was a trend in the collagen X Tg or KO murine subsets for an increase in myeloid GM and GEMM derived colonies (**Fig. 5A, B**). To further enumerate the erythroid outgrowth potential of the collagen X Tg and KO mice, pre-erythroid colonies were enumerated from marrow aspirates by growth in erythropoietin-supplemented media. Increased numbers of pre-erythroid colonies were measured from collagen X KO perinatal lethal mice, while significantly less were observed from the collagen X Tg and KO mice with a mild phenotype, all of which can be visually appreciated with marrow histology (**Figs. 1** **,** **2A**, **5C**). Additionally, these assays confirmed altered B lymphopoiesis in the collagen X Tg and KO mice, with dramatic reduction in pre-B cell colony outgrowth from the perinatal lethal subset, all of which supports the B220^+^, CD138^+^, IgD^+^, and IgM^+^ flow cytometry from marrow derived lymphocytes (**Figs. 2B,C**, **5D**) [5], [7].

**Figure 6:**
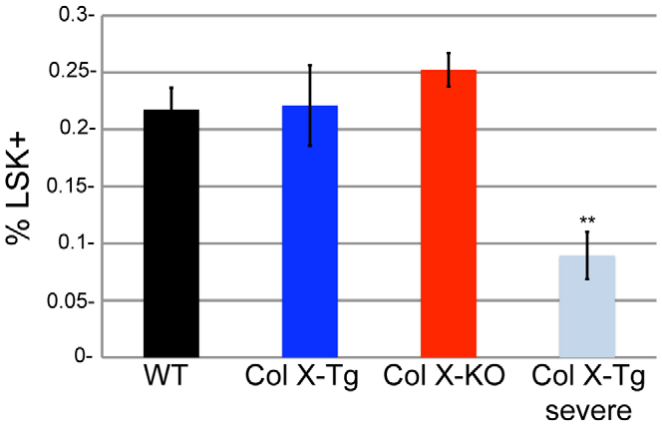
Number of LSK cells in collagen X moue bone marrow. Quantification of lineage^-^, Sca-1^+^, cKit^+^ (LSK) cells in the bone marrow of week-3 wild type (WT), collagen X transgenic (Tg), collagen X null (KO) and the perinatal lethal collagen X Tg subset (Tg-severe) via flow cytometry. 10 mice per group. (**p<0.001)

The changes in colony number measured in the CFC assays may have resulted from an altered number of hematopoietic precursor cells in the collagen X mouse. To test this, we analyzed bone marrow cells from wild type and collagen X Tg and KO mice for a hematopoietic stem cell (HSC) enriched population, lineage marker^-^, stem cell antigen-1^+^ and cKit^+^ (Lin-/Sca-1+/cKit+, LSK), which allowed comparison of the number of precursor cells from which the GM, GEMM, BFU-E and pre-B colonies could arise. No statistical difference were measured between the wild type and collagen X Tg/KO mice, however the perinatal lethal mice had a reduction in the percent LSK cells (**Fig. 6**) [19], [20]. Overall, the collagen X Tg and KO mice had sufficient numbers of precursor cells to generate GM, GEMM, BFU-E and Pre-B colonies, suggesting that the differences in colony outgrowth measured are likely due to changes in differentiation potential of the hematopoietic precursor cells.

### Serum Antibody ELISA Suggest Active Immune Response in the Perinatal Lethal Subset of Collagen X Tg and KO Mice

**Figure 7:**
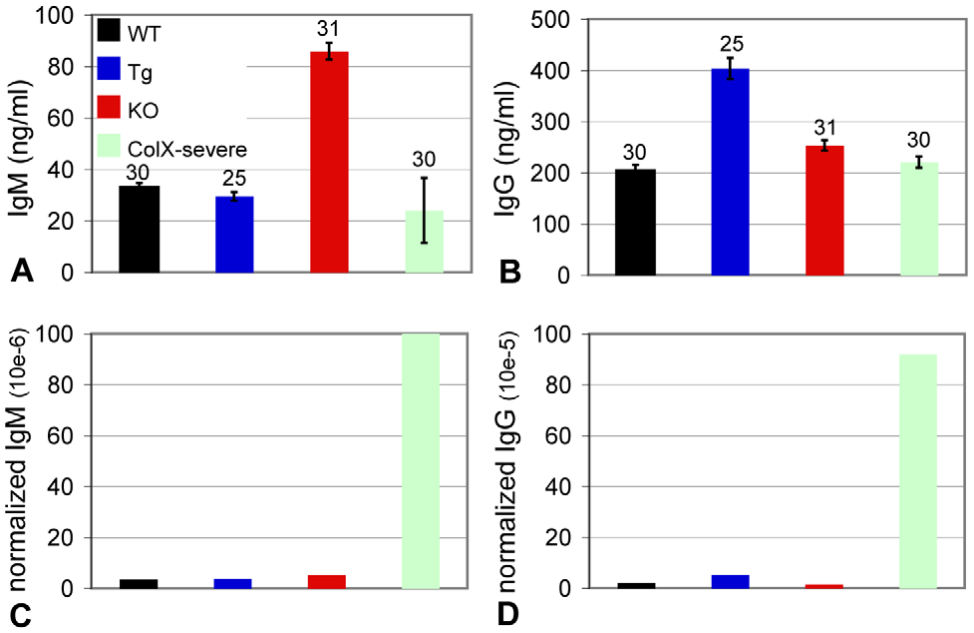
Collagen X perinatal lethal mice have increased levels of serum antibodies. Sera from week-3 wild type (WT), collagen X transgenic (Tg), null (KO) and perinatal lethal (Tg severe and KO severe; pooled) mice were assayed for IgM and IgG antibodies with ELISA. (A-B) Total IgM and IgG serum antibody measured. (C-D) Normalized levels of IgM and IgG antibodies calculated as (ng/ml of serum antibody)/(total number of live B220 splenocytes). Numbers above the standard error of the mean are number of mice per group.

The severe defect in B lymphopoiesis in the collagen X perinatal lethal mice revealed in the CFC assays (**Fig. 5D**) may lead to defects in the acquired immune response toward opportunistic infections, resulting in the observed wasting of a subset of these mice. Further, the reduction of LSK cells in the perinatal lethal collagen X Tg (**Fig. 6**) mice may indicate a mobilization of HSCs from the marrow, such as during tissue repair, inflammation or infection [19], [20]. Since activation of B lymphocytes by pathogens results first in the secretion of antigen specific IgM antibodies and then IgG antibodies, serum IgM and IgG levels were measured from week-3 collagen X Tg and KO mice as indicators of infection. Collagen X KO mice had increased levels of IgM, consistent with increased numbers of B lymphocytes (**Figs. 2B, C**, **3B, C** **,** **7A**), whereas collagen X Tg mice had elevated levels of IgG antibody, and there were no differences between the perinatal lethal subsets and wild type mice. However, the total number of B220^+^ B lymphocytes in the spleens of the perinatal lethal collagen X Tg and KO mice was drastically reduced (**Fig. 3C**). Thus, if the level of serum antibodies was normalized to the number of B lymphocytes present in the spleen, i.e. cells with the potential to secrete pathogen specific antibody, the perinatal lethal mice had considerably elevated levels of circulating IgM and IgG compared to wild type, Tg, and KO mice, consistent with an ongoing infection (**Fig. 7C, D**).

### Opportunistic Infections Cause Perinatal Lethality in Collagen X Tg and KO Mice

**Figure 8:**
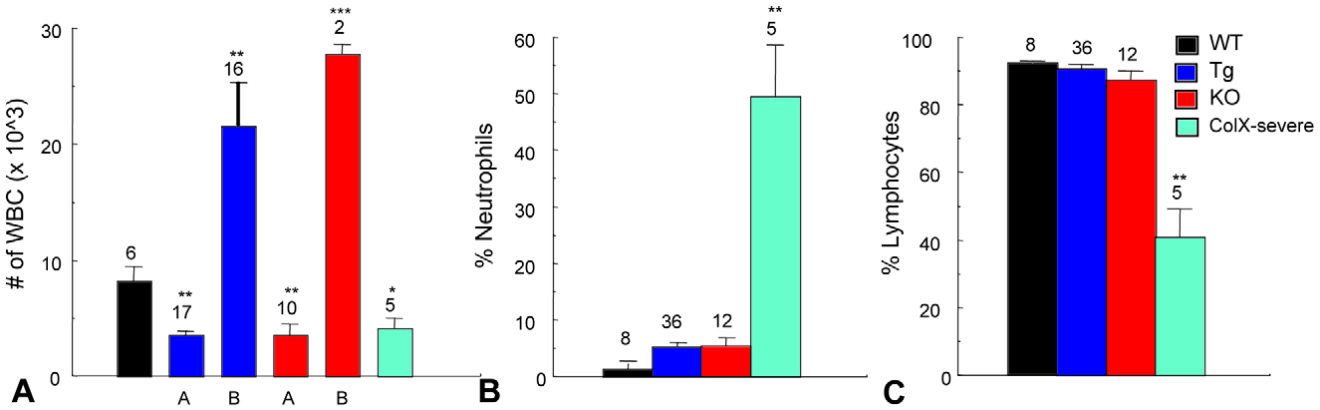
Peripheral blood complete blood count (CBC) analysis implicates infections in collagen X perinatal lethal mice. (A) White blood cell (WBC) counts from wild type (WT), collagen X transgenic (Tg), null (KO) and perinatal lethal (Tg severe and KO severe; pooled) reveal two groups of Tg and KO mice (A and B). (B) Percentage of neutrophils from peripheral blood reveals a thirteen-fold increase in the perinatal lethal subset. (C) Percentage of lymphocytes reveals a two-fold decrease in the perinatal lethal subset. Numbers above the standard deviations are number of mice per group (*p<0.05, **p<0.001).

To assess systemic infection in the perinatal lethal mice, various organs from week-3 collagen X Tg and KO mice were cultured in antibiotic-free media. The overall percentage of perinatal lethal mice with bacterial or fungal outgrowth from liver, lung, and spleen was fourteen times greater than wild type, whereas tissues from outwardly healthy collagen X Tg and KO mice were only 2 times greater (**Table 3**). Additionally, complete blood count (CBC) of peripheral blood from week-3 mice assessed levels of circulating lymphocytes and neutrophils, both involved in immune response toward infection. Total lymphocytes were decreased two-fold in the perinatal lethal mice compared to wild type cohorts, as predicted by flow cytometry and CFC assays (**Figs. 2B, C**, **3B, C**, **5D**, **8C**). Additionally, the collagen X Tg and KO mice displayed either increased (group A) or decreased (group B) levels of total white blood cells (**Fig. 8A**). Further, based on increased antibody secretion of B lymphocytes and rampant organ microbial outgrowth in the perinatal lethal mice (**Fig. 7C, D**; **Table 3**), we predicted ongoing infections, which would result in increased levels of neutrophils [21]. The CBC analysis confirmed an increase in both number of and total percentage of circulating neutrophils in the perinatal lethal mice compared to wild type (six-fold and thirteen-fold respectively), supporting this hypothesis (**Fig. 8B** and data not shown).

**Table 3:** Increased microbial contamination during In vitro organ cultures from collagen X perinatal lethal mice.

	Lung	Liver	Spleen
**WT**	8% (1/12)	0% (0/12)	0% (0/12)
**Col X Tg + KO**	6% (1/17)	6% (1/17)	6% (1/17)
**Col X severe**	42% (8/19)	42% (8/19)	31% (4/13)

Isolated and cultured lung, liver and spleen from week-3 control (WT), collagen X transgenic and null (Col X Tg + KO, pooled data), and Tg and KO perinatal lethal (Col X severe; pooled data) mice reveal increased microbial contamination in perinatal lethal samples. Percent contamination was determined as number of wells contaminated with either bacteria or fungus after 24 hr culture, over the total number of wells per mice tested (in parenthesis).

## Discussion

The skeleto-hematopoietic disease phenotype of the collagen X Tg and KO mice has highlighted an intimate link between endochondral skeletal elements, specifically growth plate hypertrophic chondrocytes and trabecular bone, and blood cell differentiation within the marrow. To directly implicate collagen X disruption within the chondro-osseous junction as the sole underlying factor of all hematopoietic and immune response changes in the mice, it was necessary to exclude not only transgene insertional mutagenesis and mis-expression of endogenous and transgenic collagen X [6], [10], [13], but also strain specific genetic modifications as possible contributors to the disease phenotype. The generation of C57Bl/6 and DBA/2J collagen X Tg and KO congenic strains revealed conservation of all the characteristic disease phenotypes observed in the outbred collagen X Tg and KO mice, thus excluding the contribution of strain specific modifiers to the disease phenotype. These data are consistent with the disruption of collagen X function at the chondro-osseous junction, namely the altered hypertrophic cartilage pericellular matrix [11] and the developing trabecular bone, as being the primary defects leading to changes within the chondro-osseus/marrow environment where hematopoietic cells develop and differentiate (**Fig. 1**). These initial changes in the chondro-osseous junction thus likely affect the marrow hematopoietic niche, which in turn may contribute to the downstream effects on lymphopoiesis and lymphatic tissue engraftment in the periphery. In support, thymus and spleen architecture and cell content are altered in the collagen X Tg and KO mice (**Figs. 3**, **4**). This could be explained by altered hematopoietic differentiation leading to, for example, decreased early T lineage progenitor production in the marrow that resulted in a hypocellular cortex and diminished immature T lymphocytes in the thymus (**Fig. 4**). Further, every Tg and KO mouse exhibits an altered B lymphocyte profile in both the marrow and spleen (**Figs. 2** **,** **3**). Moreover, flow cytometry and CFC assays showed decreased levels of B lymphocytes in Tg and perinatal lethal collagen X mice at week-3 and transiently increased levels in KO mice (**Table 1**; **Figs. 2**, **3**, **5D**). These differences in B lymphocyte profiles may indicate an enhanced ability of the KO mice to cope with insults on their immune system (e.g. opportunistic infections), and thus may account for reduced perinatal lethality at week-3 and overall less severe phenotype compared to the Tg strains. Of further consideration, lymphopoietic changes persist throughout life in all collagen X mice, which may relate to lack of growth plate closure in rodents due to continued EO. Overall, these data link changes in EO to altered lineage commitment of collagen X mouse hematopoietic cells, though not total numbers of precursor cells (**Fig. 6**), and implicate the marrow environment of the collagen X Tg and KO mice as being impaired in its ability to support lymphopoiesis, resulting in defective immune responses throughout life [8].

Data presented here implicate the disruption of collagen X function as the cause of the phenotypic spectrum of disease severity manifestation in mice. Our studies suggest that the most severe manifestation of the disease phenotype, namely perinatal lethality, may ensue from opportunistic infections and deregulated immune responses. Specifically, the perinatal lethal mice have increased levels of circulating IgM and IgG antibodies, increased incidence of organ bacterial and fungal outgrowth in vitro, and increased levels of serum neutrophils (**Table 3** **;** **Figs. 7**, **8B**), all of which are indicators of an ongoing infection [21]. Moreover, Sulfatrim® antibiotic treatment of pregnant and nursing collagen X females and oxytetracycline injections into mice displaying perinatal lethality resulted in ablation of the moribund phenotype (unpublished observation). Additionally, the perinatal lethal mice have significantly diminished levels of B and T splenocytes, thymocytes and T regulatory cells, suggesting that perinatal lethality may ensue from infection and a deregulated immune response [8]. Further, the immune response in all collagen X Tg and KO mice at every age is defective, as observed with in vivo parasite challenges, in vitro splenocyte stimulation with non-specific mitogens and CBC analyses indicating abnormal (significantly higher or lower) levels of total white blood cells (**Fig. 8A** and [8]). Together, these data further underscore that initial changes at the chondro-osseous junction lead to defects in lymphopoiesis, peripheral lymphatic tissue establishment and immune responses.

**Figure 9:**
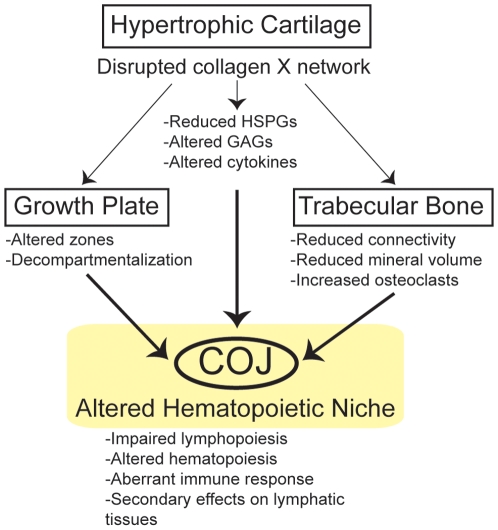
All collagen X Tg and KO mice have skeleto-hematopoietic defects that directly arise from collagen X disruption in hypertrophic cartilage. Disruption of collagen X function in hypertrophic cartilage of the growth plate results in the loss of a collagen X-containing pericellular network, leading to a reduction and altered compartmentalization of heparan sulfate proteoglycans (HSPGs) within the chondro-osseous junction (COJ) [11], as well as altered cytokine production by COJ cells ([8] and unpublished data). Resultant skeletal defects involve all EO-derived skeletal elements, and manifest as compressed growth plates [5], and reduction in the connectivity, mineral volume and overall amount of trabecular bone (**Fig. 1** and unpublished data). Collectively, these changes culminate in an altered COJ, which affects the differentiation of resident hematopoietic stem cells, thereby negatively effecting lymphopoiesis and immunity. Hematopoietic defects manifest as impaired lymphopoiesis in the marrow (**Fig. 2**), with subtle effects on other blood cell lineages (**Fig. 5**). Secondary hematopoietic/immune defects, arising as a consequence of an altered marrow environment, involve an impaired lymphocyte population in all lymphatic organs (**Figs. 3**, **4**), and impaired immune response throughout life [8].

In summary (**Fig. 9**), these data indicate that all Tg and KO mice with disrupted collagen X function have unique skeletal and hematopoietic defects that arise from collagen X disruption in hypertrophic cartilage. Skeletal defects involve all EO-derived skeletal elements, and manifest as altered growth plates and reduced trabecular bone (**Fig. 1**; [5], [7]). Hematopoietic defects manifest as impaired lymphopoiesis in the marrow (**Fig. 2**), coupled with increased myelopoiesis (**Fig. 5**). While it is still unclear whether these changes in the marrow ensue as a direct or an indirect consequence of collagen X impairment, we envision them contributing to the secondary hematopoietic/immune defects, which involve an impaired lymphocyte population in all lymphatic organs (**Figs. 3**, **4**), and impaired immune response throughout life [8].

An emerging provocative hypothesis from this study suggests that disruption of collagen X function in the EO-derived chondro-osseous environment may alter the hematopoietic niche. The chondro-osseous junction includes hypertrophic chondrocytes, trabecular bone, osteoclasts, the endosteum, marrow and stromal constituents, and associated ECMs (**Fig. 1**). Thus, it is plausible that changes in the well orchestrated events of EO that affect the hypertrophic chondrocyte-to-trabecular bone transition may likewise affect the unique marrow environment, leading to hematopoietic/immune disorders. Support can be found in human “immuno-osseous disorders” that link skeletal, hematopoietic and immune defects, and include: cartilage-hair hypoplasia (CHH), Kostmann's syndrome, Shwachman-Diamond syndrome, Schimke dysplasia, Fanconi anemia, Diamond-Blackfan anemia, Dubowitz, Omenn and Barth syndromes, kyphomelic dysplasia, spondylo-mesomelic-acrodysplasia, and adenosine deaminase deficiency [22], [23], [24], [25], [26], [27], [28], [29], [30], [31], [32], [33], [34], [35], [36]. For example, CHH patients, with mutations in the RNA component of the ribonucleoprotein complex RNase MRP, present with disproportionate short stature and deficient cellular immunity [28], [37], reminiscent of the collagen X murine metaphyseal dysplasia and hematopoietic defects. Additionally, CHH patients display altered levels of several immune mediators, as is reported with the collagen X mice [8]. Even though the genetic basis for some of these disorders does not include obvious players in skeletogenesis or ECM establishment and maintenance, it is conceivable that similar pathways altered in the collagen X Tg and KO mice may also be affected, leading to the similar disease phenotypes.

Recently, at least two distinct HSC-supportive niches have been described in the marrow: an osteoblastic niche, ascribed to osteoblasts residing on bone surfaces [38], [39], [40], [41] and a vascular niche, ascribed to endothelial cells lining sinusoids [42], [43], [44], both of which may encompass the lymphopoietic niche as well [45], [46]. For example, the osteoblast niche has been shown to support hematopoietic development, especially regulation of B lymphopoiesis. Specifically, Emerson and colleagues demonstrated in vitro and in vivo that osteoblasts are essential for B-cell commitment and maturation [47], [48], and Scadden and Kronenberg's groups demonstrated that this ability may be mediated by G_s_α-dependent signaling pathways [49]. Moreover, Scadden's group has shown that HSCs home to and are retained at calcium-enriched regions where bone is being remodeled, via a calcium-sensing receptor [50]. On the other hand, Sacchetti et al. have shown that osteoblasts alone cannot recapitulate a hematopoietic niche upon transplant, and implicated a subpopulation of bone marrow stromal cells, the subendothelial cells within sinusoidal walls in marrows, as competent to generate osteogenic progenitors and to recapitulate a hematopoietic marrow upon transplant [43]. Taken together, these data may be interpreted by associating the postulated “niches” to cells and ECM environments that undergo or arise as a consequence of EO.

Our group was the first to propose that EO establishes the specific microenvironment in the marrow for hematopoietic cell development, which was based on the unique skeleto-hematopoietic defects in mice with disrupted collagen X function [6]. Since collagen X expression is restricted to hypertrophic cartilage, this directly implicates the chondro-osseous junction in contributing to the hematopoietic niche [5], [8], [51]. Recent data from two other groups directly support our hypothesis by implicating EO-progenitors in establishment of a hematopoietic marrow. Specifically, Akintoye et al. demonstrated that transplantation of marrow stromal cells from EO-derived sites can recapitulate a chondro-osseous junction with a marrow, while IO-derived cells only generate dense, marrow-free bone [52]. Likewise, Chan et al. confirmed these observations by sorting for subpopulations of either EO or IO-progenitors from marrow and demonstrating that upon transplantation only the EO-progenitors could recapitulate an HSC niche [53].

The contributions that ECM may have on hematopoiesis are still underappreciated. Hematopoietic cells preferentially localize to bone surfaces where bone matrix, osteoblasts, osteoclasts, and marrow meet [54], [55]. Here, these components, together with stromal cells, vascular cells, and various ECM components (collagens, glycosaminoglycans (GAGs), proteoglycans (PGs), glycoproteins) [56] encounter to compartmentalize the marrow. The ECM network not only defines the tissue's mechanical properties, but also may mediate hematopoietic cell behavior by sequestering bioactive factors such as cytokines and growth factors. Such interactions between the physical components and diffusible factors likely comprise a hematopoietic niche. A prominent example of such interactions involves heparan sulfate proteoglycans (HSPGs), which are proposed to orchestrate hematopoietic cell niches by regulating cytokine and growth factor bioactivity and availability for hematopoietic cells [57], [58], [59], [60], [61]. It is noteworthy that we have shown that the primary consequence of collagen X disruption within the condro-osseous junction is the loss of a pericellular network, likely consisting of collagen X in hypertrophic cartilage [11], resulting in a decompartmentalization of HSPGs and GAGs such as hyaluronan [11]. It may also be relevant that a number of HSPG binding cytokines that are involved in hematopoiesis, immune cell development, differentiation, and response [62], [63], [64], [65] are miss-expressed in the collagen X mice [8], and thus may affect lymphopoiesis and immunity.

Overall, we envision that the collagen X/HSPG/GAG network sequesters hematopoietic cytokines and growth factors at the chondro-osseous junction for chondrocyte, osteoblast, stromal cell, and hematopoietic signaling. These cytokines can be liberated along trabecular bone surfaces upon remodeling, and thereby may promote hematopoietic differentiation. Such a hematopoietic niche at the chondro-osseous junction is likely transient, due to remodeling of the hybrid trabecular bone-hypertrophic cartilage spicules into mature secondary bone by osteoclasts, as also suggested by Scadden and colleagues [50]. The continual turnover of this tissue may result in liberation of HSPG-bound cytokines to the local environment, exactly where hematopoietic cells have been reported to reside [50], [66], [67], [68]. Conversely, a disruption of this unique environment would result in altered hematopoiesis, as seen in the collagen X Tg and KO mice. In support, recent bone marrow transplantations in neonatal collagen X Tg mice implicate the defective chondro-osseous environment, rather than alterations in hematopoietic precursors, such as trafficking or homing issues, as the locus for the observed hematopoietic defects (unpublished data). Therefore, disruption of the collagen X/HSPG/GAG network at the chondro-osseous junction in the collagen X Tg and KO mice may impact sequestration of hematopoietic cytokines and growth factors necessary for hematopoietic cell differentiation. This model would explain the altered skeletal and hematopoietic/immune phenotype of the collagen X Tg and KO mice, and may likewise provide a link for certain human immuno-osseous or hematopoietic disorders.

## Materials and Methods

### Mouse Generation, Maintenance and Organ Cultures

All animals were handled in strict accordance with good animal practice as defined by the University of Pennsylvania Institutional Animal Care and Use Committee, and all animal work was approved by the Animal Welfare Committee at the University of Pennsylvania. Congenic mice were generated by inbreeding females from either two outbred collagen X Tg lines (1.6–293Δ and 4.7–21Δ, strain: C57BL/6, SJL and DBA/2J) or from one outbred collagen X KO (strain: C57Bl/6 and 129Sv) line, over 12 generations to a limited number of C57BL/6 and DBA/2J males, and then interbreeding to establish homozygosity [16]. Genotypes were established via PCR and Southern Blot [6], [7], [10]. This resulted in six congenic mouse strains: 1.6–293Δ–B6, 1.6–293Δ–DBA, 4.7–21Δ–B6, 4.7–21Δ–DBA, KO-B6, and KO-DBA.

Mice were maintained aseptically in a barrier facility and inspected daily for growth, behavioral, skeletal, or hematopoietic abnormalities [5]. Characteristic features of perinatal lethality around week-3 (usually days 19–21, but occasionally within days 16–30) include reduced size, back hunching, lethargy, mobility changes, and wasting. Perinatal lethal ratios were calculated as number of perinatal lethal ∼week-3 mice divided by total number of week-3 mice. Assays with the perinatal lethal subset were performed prior to demise of the mice. For organ outgrowth assays, tissues from wild type (WT), collagen X Tg and KO mice were aseptically harvested, cut in half for duplicates, placed in 12 well tissue culture plates (TKR Biotech products) with 1 ml sterile RPMI media with 10% FBS (Gibco), incubated at 37°C for 3–6 days, and visually screened for contamination every 12 hr.

### Histology

Hind limbs and lymphatic organs from week-3 WT and collagen X Tg and KO mice from the outbred and congenic lines with mild (survivor) and perinatal lethal phenotypes were fixed in 4% formaldehyde/phosphate-buffered saline (PBS) (pH 7.4) at 4°C for 1 wk. Hind limbs were decalcified overnight in 4% formalin, 1% sodium acetate, 10% EDTA. All samples were dehydrated in ascending ethanol series, cleared with Propar (Anatech), and paraffin-embedded. Six-micron sections were stained with Giemsa or Hematoxylin and Eosin (Sigma).

For cryosectioning, tissues were embedded in Tissue Tek OCT (Sakura Finetek), frozen in ethanol/dry ice, and cut to 6–8 µm sections, which were acetone-fixed (1 min), rinsed in PBS (Sigma, 2×8 min), and incubated in 0.3% H_2_O_2_/PBS (10 min for spleen; 5 min for thymus). After blocking (4% heat-inactivated FBS, in PBS; 30 min, Sigma), tissues were incubated with anti-mouse primary antibodies (10 mg/ml in block, 60 min, room temperature, RT), including CD45R/B220, Ter119 and isotype controls (purified rat IgG2a kappa, IgG2b kappa, BD PharMingen). After rinses (with block, 4×8 min), biotinylated anti-rat IgG (5 mg/ml) secondary antibodies (Vector Labs, in block, 30 min, RT) were added, then incubated in Vectastain ABC Reagent (Vector Labs, 30 min, RT) containing avidin and biotinylated HRP, and rinsed (with PBS, 4×8 min). Diaminobenzidinetetrahydrochloride solution (Pierce Chemical Co., 5 min) was used to visualize the reaction. Sections were mounted with Aqua-mount (Lerber Labs) and viewed with an Olympus BX60 light microscope. Data was recorded with the Spot Flex digital camera, Spot software and brightness/contrast was adjusted with Adobe Photoshop CS.

### Colony Forming Cell Assay

Colony forming cell assays involved the murine protocol from Stem Cell Technologies. Briefly, tibial and femoral marrow was flushed from week-3 WT, collagen X Tg and KO mice with PBS, red cells were lysed (ACK buffer: 0.15M NH_4_Cl, 10 mM KHCO_3_, 0.1 mM Na_2_EDTA, pH 7.4, 1 min), and remaining cells plated in 35 mm dishes at: 2×10^4^/plate in complete Methocult™, 2×10^5^/plate in erythropoietin enriched Methocult™, and 1×10^5^/plate in IL-7 enriched Methocult™(Stem Cell Technologies). These different media support and stimulate the growth of erythroid blast (erythropoietin enriched, 3 days), granulocyte-macrophage and granulocyte-erythroid-monocyte-macrophage (complete, 6 days) or pre-B lymphocyte (IL-7 enriched, 7 days) colonies. Cultures were incubated at 37°C, 5% CO_2_, and quantified based on morphological recognition using a light microscopy as described by Stem Cell Technologies. Results were plotted in Excel and two-tailed, two sample unequal variance T-tests were used to establish significance.

### Peripheral Blood Analysis

Peripheral blood was isolated via heart puncture using heparinized micro-hematocrit capillary tubes (Fisher) and submitted to the Special Species Clinical Pathology core at Univ. Penn. Sch. Vet. Med. for complete blood count. Data was plotted in Excel and significance was established as described above.

### Antibody ELISA

Antibody ELISAs were performed according to Bethyl Labs Inc. Briefly, blood from heart puncture was clotted at RT, and serum isolated via centrifugation (6000 rpm, 10 min, 4°C). Levels of IgG and IgM were assessed using a mouse specific IgG and IgM kit (Bethyl Labs Inc.) and developed with TMB Peroxidase Substrate and Peroxidase Solution B (Kirkegaard and Perry). Plates were read at 450 nm on a Biorad Benchmark plus. To calculate the level of serum IgG and IgM a standard curve was generated as per the Antibody ELISA kit protocol. Results were plotted in Excel and significance was established as described above.

### Flow Cytometry

Flow cytometry was performed as previously described [5]. Briefly, tibial and femural marrows were collected and erythrocytes were lysed (0.17 mol/L Tris, 0.16 mol/L NH4Cl). Spleens and thymuses were homogenized in PBS via a Tenbroeck Tissue Grinder (Wheaton). Cells were labeled with primary antibodies (0.2 µg/10^6^ cells): anti-CD4/L3T4, CD8a/Ly-2, CD45R/B220, IgM, IgD, and isotype controls, followed by FITC polyclonal anti-rat IgG secondary antibodies (0.1 µg/10^6^ cells). Propidium iodide (PI; 10 µl at 50 µg/ml) was added for dead cell exclusion. For LSK analysis, bone marrow cells were Fc blocked then stained with mouse lineage antibody cocktail (APC), anti-mouse cKit (FITC) and anti-mouse Sca-1 (PE). All antibodies were purchased from BD Pharmingen. The FACS Calibur was used with CELL Quest 3.1 (Becton Dickinson) for data acquisition and analysis.
